# Osmotic adjustment and hormonal regulation of stomatal responses to vapour pressure deficit in sunflower

**DOI:** 10.1093/aobpla/plaa025

**Published:** 2020-06-19

**Authors:** Amanda A Cardoso, Timothy J Brodribb, Cade N Kane, Fábio M DaMatta, Scott A M McAdam

**Affiliations:** 1 School of Biological Sciences, University of Tasmania, Hobart, Tasmania, Australia; 2 Departamento de Biologia Vegetal, Universidade Federal de Viçosa, Viçosa, Minas Gerais, Brazil; 3 Purdue Center for Plant Biology, Department of Botany and Plant Pathology, Purdue University, West Lafayette, IN, USA

**Keywords:** Abscisic acid, leaf osmotical potential, leaf turgor loss point, stomatal closure, vapour pressure deficit, wilty mutant

## Abstract

Dynamic variation of the stomatal pore in response to changes in leaf–air vapour pressure difference (VPD) constitutes a critical regulation of daytime gas exchange. The stomatal response to VPD has been associated with both foliage abscisic acid (ABA) and leaf water potential (Ψ _*l*_); however, causation remains a matter of debate. Here, we seek to separate hydraulic and hormonal control of stomatal aperture by manipulating the osmotic potential of sunflower leaves. In addition, we test whether stomatal responses to VPD in an ABA-deficient mutant (*w-1*) of sunflower are similar to the wild type. Stomatal apertures during VPD transitions were closely linked with foliage ABA levels in sunflower plants with contrasting osmotic potentials. In addition, we observed that the inability to synthesize ABA at high VPD in *w-1* plants was associated with no dynamic or steady-state stomatal response to VPD. These results for sunflower are consistent with a hormonal, ABA-mediated stomatal responses to VPD rather than a hydraulic-driven stomatal response to VPD.

## Teaser

In sunflower a plant hormone called abscisic acid closes the microscopic pores on the leaves to prevent excessive water loss when humidity decreases.

## Introduction

Stomata on the leaves of terrestrial plants regulate the diffusion of CO_2_ and water vapour between the leaf and the atmosphere, thereby controlling plant hydration and photosynthetic rate (Farquhar and Sharkey 1982). Dynamic regulation of the stomatal pore therefore forms one of the primary controllers of atmospheric water and CO_2_ fluxes, as well as dictating the efficiency with which plants use water (Lawson and Blatt 2014), and the operational safety of plants with regard to avoiding damaging desiccation (Brodribb and McAdam 2017). The most pervasive stomatal dynamics are responses to light and leaf–air vapour pressure difference (VPD). Light responses are tied to the direct action of membrane-bound phototropins in guard cells and an integrated photosynthetic signal (Shimazaki *et al.* 2007), while responses to VPD appear to be produced by changes in leaf hydration (Mott and Parkhurst 1991). Stomatal responses to VPD are a critical determinant of the efficiency of water use by leaves and are the focus of this study.

Stomata close as VPD increases, thereby substantially moderating the impact of increased evaporative demand on leaf water loss. The mechanism responsible for this response in angiosperms remains under debate, with some research supporting the involvement of passive changes in guard cell turgor as the primary driver of these responses (i.e. passive-hydraulic regulation) (Mott *et al.* 1997; Assmann *et al.* 2000; Mott and Peak 2013; Peak and Mott 2011). This literature is largely based on the predictability of steady-state stomatal responses to changes in VPD using biophysical models that assume guard cell turgor changes passively in response to altered VPD. Alternatively, there is an argument that stomatal responses to changes in VPD in angiosperms are primarily caused by ion fluxes in the guard cell, mediated by a metabolic signal (Bunce 1996; Bauerle *et al.* 2004; Bauer *et al.* 2013; McAdam and Brodribb 2015; Merilo *et al.* 2018). There is little consensus as to which of these mechanisms are primarily responsible for regulating stomatal responses to changes in VPD in angiosperms **[see****Supporting Information—Table S1****]**. Understanding this mechanism is of considerable importance because attempts to increase the productivity of irrigated crops have identified VPD responses as a primary target for improvement (Sinclair *et al.* 2016).

One proposal is that stomatal responses to VPD in angiosperms are regulated by the action of the phytohormone abscisic acid (ABA) (Grantz 1990; Xie *et al.* 2006; Bauer *et al.* 2013; McAdam and Brodribb 2015). Suggestions of ABA as the driver of stomatal responses to VPD (Bunce 1996) have received support from studies correlating responses to VPD with changes in hormone levels in leaves (Bauerle *et al.* 2004; Giday *et al.* 2013; McAdam and Brodribb 2015; Qiu *et al.* 2017), as well as observations of reduced responses to changes in VPD in ABA biosynthetic and signalling mutants (Xie *et al.* 2006; Bauer *et al.* 2013). More recent work shows that the upregulation of ABA biosynthetic genes driven by changes in leaf turgor or cell volume occur in a time frame of minutes, providing sufficiently fast activity to explain the relatively rapid closing responses of stomata to step increases in VPD (McAdam *et al.* 2016; Sussmilch *et al.* 2017). While there is evidence of stomatal responses to changes in VPD in angiosperms being driven by ABA, there are published observations of stomatal closure in some *Arabidopsis* ABA-deficient and ABA-insensitive mutants in response to a step change in VPD from 0.4 to 1.3 kPa (Assmann *et al.* 2000). Recent reports of such behaviour have re-opened a discussion regarding the role of ABA levels in regulating stomatal responses to VPD in angiosperms (Merilo *et al.* 2018).

Merilo *et al.* (2018) found that while ABA-deficient mutants may have a stomatal response to changes in VPD, mutants in the key ABA signalling gene *OPEN STOMATA1* (*OST1*) (that encodes a protein kinase which activates the guard cell SLAC1 Cl^−^ channel in response to ABA; Jezek and Blatt 2017) do not respond to changes in VPD. These results have been incorporated into a new theory whereby ABA defines a background stomatal conductance while OST1 gates the sensitivity of membrane transporters to a step increase in VPD (Pantin and Blatt 2018). The validity of reported stomatal sensitivity to changes in VPD in ABA-deficient mutants is challenged by models and isotope analyses that indicate subsaturation of water vapour in the substomatal cavity occurs at high VPD (Vesala *et al.* 2017; Cernusak *et al.* 2018; Buckley and Sack 2019). An apparent stomatal closure in response to increasing VPD in ABA signalling mutants of *Populus* does not exist when gas exchange measurements are corrected for subsaturation of water vapour in the substomatal cavity (Cernusak *et al.* 2019). Given these results, there is the high possibility that in plants with low leaf hydraulic conductance, like *Arabidopsis* (Caringella *et al.* 2015), an apparent stomatal closure (measured by leaf gas exchange) at high VPD can be observed in ABA biosynthesis or signalling mutants because open stomata lead to a rapid subsaturation of water vapour in the substomatal cavity and the underestimation of stomatal conductance.

Given that foliage ABA biosynthesis is likely triggered by changing cell volume or membrane–cell wall interactions as leaf turgor declines close to zero (Zabadal 1974; Beardsell and Cohen 1975; Pierce and Raschke 1980; Davies *et al.* 1981; Creelman and Zeevaart 1985; McAdam and Brodribb 2016; Sack *et al.* 2017), if ABA does mediate the stomatal responses to VPD we would hypothesize that stomatal closure at high VPD will occur when leaf water potential (Ψ _*l*_) declines sufficiently close to the water potential of turgor loss point (Ψ _tlp_), thereby triggering ABA biosynthesis. Osmotic adjustment, which reduces Ψ _tlp_, provides an excellent experimental system with which to test whether a shift in the trigger for ABA biosynthesis alters stomatal sensitivity to changes in Ψ _*l*_. Here, we manipulated turgor loss point by inducing osmotic adjustment in sunflower (*Helianthus annuus*) and monitored Ψ _*l*_, foliage ABA levels and the stomatal responses to a step increase in VPD. In addition, we tested whether stomatal responses to VPD in a classical ABA-deficient mutant (*w-1*) of sunflower are like the wild-type (Fambrini *et al.* 1994, 1995). Sunflower was selected because it can osmotically adjust in response to high VPD and soil water deficit (Turner *et al.* 1978; Chimenti *et al.* 2002; Cardoso *et al.* 2018), and has a high leaf hydraulic conductance (Guyot *et al.* 2011), which might overcome the limitation of water supply to open stomata at high VPD, thereby reducing the effect of potential subsaturation of water vapour in the substomatal cavity (Vesala *et al.* 2017; Cernusak *et al.* 2018; Buckley and Sack 2019).

## Materials and Methods

### Plant material and growth conditions

Individuals of sunflower cv. Yellow Empress (Asteraceae) were grown for *c.* 60 days under two contrasting conditions, i.e. well-watered and water-limited. Well-watered plants were grown inside a controlled glasshouse regulated at 16-h day at 25 °C/15 °C day/night temperatures, VPD at *c.* 1.0 kPa during the day and natural light [maximum photosynthetic photon flux density (PPFD) of 1500 µmol m^−2^ s^−1^ at the pot surface]. Plants were grown in *c*. 3-L plastic pots filled with potting mix and watered daily to full capacity resulting in mean predawn water potential of −0.18 ± 0.04 MPa and midday water potential of −0.55 ± 0.07 MPa.

**Figure 1.**
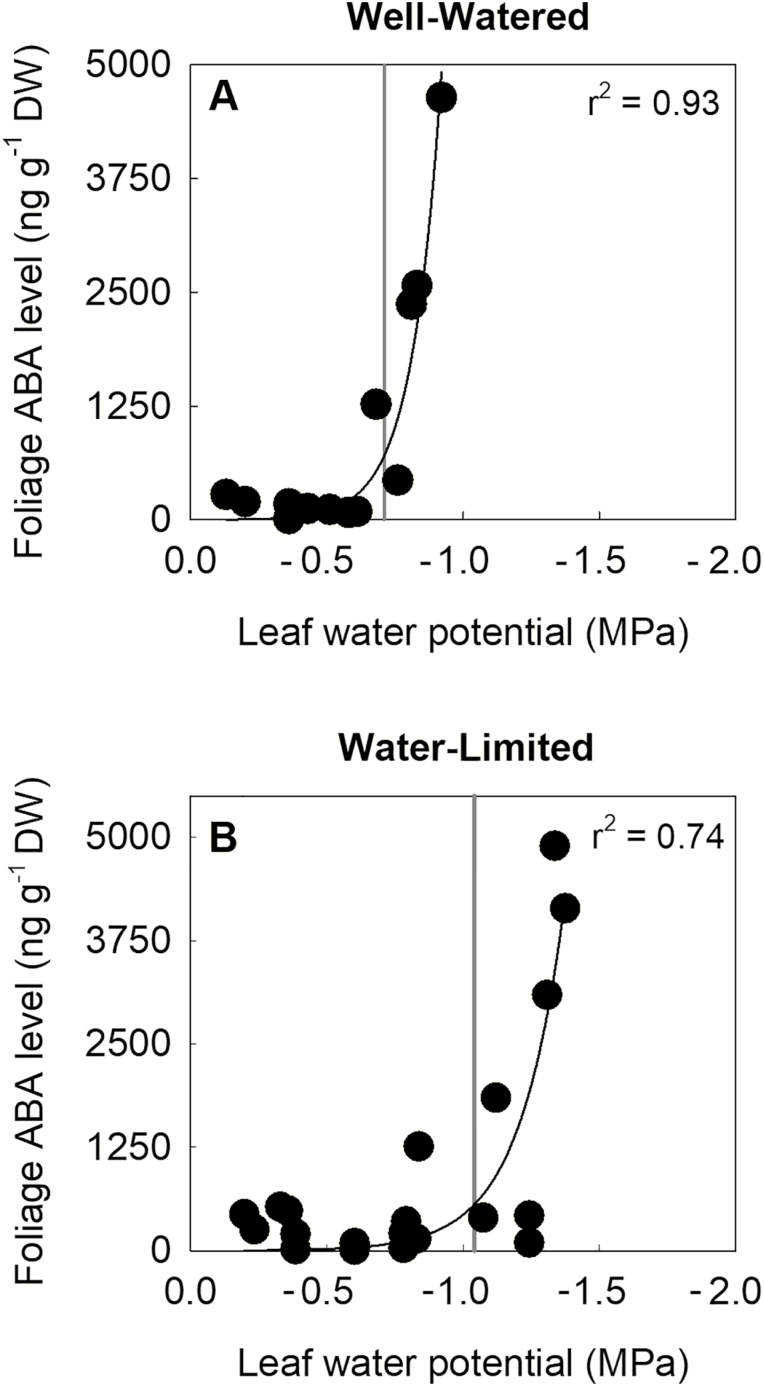
The relationship between foliage ABA level and leaf water potential (*n* = 3) collected in bench-dried branches of sunflower cv. Yellow Empress plants that were grown under either well-watered (A) or water-limited (B) conditions. Vertical grey lines indicate water potential at turgor loss point (mean; *n* = 3; see Table 1).

Water-limited plants were grown outside the glasshouse during summer under a natural *c.* 16-h day at *c.* 23 °C/13 °C day/night temperatures, an average daily VPD of 1.45 ± 0.7 kPa, and natural light (maximum PPFD of 1800 µmol m^−2^ s^−1^ at the pot surface). Plants were grown in *c*. 3-L plastic pots filled with potting mix and watered three times per week to full capacity causing cycles in predawn and midday water potential **[see****Supporting Information—Fig. 1****]**. At the end of 60 days, both well-watered and watered-limited plants were *c.* 100–120 cm tall and each plant had *c.* 20 leaves. All measurements were carried out using leaves that had expanded during the treatment period.

### Leaf osmotic potential and turgor loss point

Three individuals for each growth condition were used to assess leaf osmotic potential at full turgor and leaf turgor loss point. Before sampling the plants, they were watered, bagged with wet paper towels and maintained in the dark overnight to ensure full turgor at the beginning of the experiments. Measurements of leaf osmotic potential were carried out using a stem psychrometer (PSY1, ICT International, Armidale, Australia) in a similar way to Bartlett *et al.* (2012). Leaf discs of *c.* 5 mm diameter were sampled from hydrated leaves, wrapped in tinfoil, and immediately frozen in liquid nitrogen to disrupt cell walls and eliminate turgor pressure. Midribs and large veins were avoided in selecting the leaf discs. The tissues were then sealed in a stem psychrometer, and the osmotic potential (Ψ _*l*_ reported by the psychrometer is here considered to be the leaf osmotic potential due to absence of turgor pressure) logged every 10 min until stable (*c.* 30 min).

The turgor loss point was determined by the relationship between Ψ _*l*_ and the water volume in the leaf (pressure–volume analysis; Tyree and Hammel 1972). Leaves were cut under water and rehydrated overnight until Ψ _*l*_ was > −0.1 MPa. Leaf weight and Ψ _*l*_ were measured over time during slow desiccation on the laboratory bench until Ψ _*l*_ began to rise due to cell damage, at least four points were collected before and after turgor loss point for each leaf. The turgor loss was determined as the point of inflection between the linear (pre-turgor loss) and non-linear (post-turgor loss) portions of the relative water content and Ψ _*l*_ relationship.

### Foliage ABA accumulation

In order to determine the relationship between Ψ _*l*_ and foliage ABA levels we monitored the ABA levels in excised leaves during slow desiccation, given leaves are the main site of ABA biosynthesis in the plant (McAdam and Brodribb 2018; Zhang *et al.* 2018). For each growth condition, three plants were watered, bagged with wet paper towels and maintained in the dark overnight to ensure full turgor at the beginning of the experiments. Early in the morning, one leaf from each individual was excised and left to dry slowly on a bench at 22 °C and low light. During the course of the next 12 h, Ψ _*l*_ and ABA levels were assessed five times in the same leaf. Leaf discs of *c.* 5 mm diameter were sampled, enclosed in a stem psychrometer and the Ψ _*l*_ was logged every 10 min until stable (*c.* 90 min). Immediately after sampling the leaf for Ψ _*l*,_ harvesting of leaf tissue for ABA levels was undertaken. An area totalling ~20 % of each leaf was removed for this experiment.

For foliage ABA assessment, leaf samples were weighed (± 0.0001 g; MS204S, Mettler-Toledo, Greifensee, Switzerland), immediately covered with cold (−20 °C) 80 % (v/v) methanol in water with 250 g L^−1^ (m/v) of added butylated hydroxytoluene, and stored at −20 °C. Samples were purified and foliage ABA levels were then quantified by physicochemical methods with an added internal standard by ultra-performance liquid chromatography tandem mass spectrometry according to McAdam (2015). Finally, the relationship between foliage ABA level and Ψ _*l*_ was fitted and the equation obtained using the curve-fitting function of the Sigma Plot software (Systat Software Inc., San Jose, CA, USA).

### VPD transitions of whole individuals

Transitions in VPD were conducted by exposing intact plants to a rapid, step increase in VPD in growth cabinets. All plants were watered and acclimated overnight in a custom-built chamber under darkness and low VPD conditions [0.75 ± 0.3 kPa (28 °C and 80 % relative humidity)]. During the next morning, starting at 0800 h, plants were illuminated with a PPFD of *c.* 300 μmol m^−2^ s^−1^, and after *c*. 90 min leaf gas exchange, Ψ _*l*_ and foliage ABA levels were measured at this low VPD condition. Plants were immediately transferred to a second adjacent growth chamber under high VPD [3.25 ± 0.3 kPa (28 °C and 14 % relative humidity), all other conditions maintained the same as the initial chamber] with the low humidity sustained by a condensing dehumidifier (SeccoUltra 00563, Olimpia-Splendid, Gualtieri, Italy). The Ψ _*l*_ was assessed at 5 and 60 min after the increase in VPD; leaf gas exchange logged every 20 min until 60 min; and leaf tissue harvested for foliage ABA analysis 60 min after the transition between chambers. The relative humidity of the air was monitored every 30 s during the experimental period using a humidity probe (HMP45AC, Vaisala, Helsinki, Finland). Air and leaf temperature were measured using a thermocouple shielded from solar radiation and connected to a data logger (CR800, Campbell Scientific, Logan, UT, USA).

For each growth condition, three individuals were used for each VPD transition. One fully expanded leaf per plant was selected for the gas exchange measurements which were performed using a portable gas analyser (GFS-3000, Heinz Walz, Effeltrich, Germany). Conditions in the cuvette were controlled at a temperature of 30 °C, 390 μmol CO_2_ mol^−1^ air, PPFD of 1000 μmol m^−2^ s^−1^ at the leaf surface and VPD was maintained at ambient VPD. One leaf per plant was randomly sampled for Ψ _*l*_ at each measurement time. After harvesting, leaves were wrapped in wet paper towel, bagged and placed in an ice box for Ψ _*l*_ measurements using a Scholander pressure chamber.

The adjacent leaf to the one used for gas exchange measurements was harvested for foliage ABA quantification. The same leaf was harvested (*c.* 5 cm^2^) at different measurement times to avoid age differences in ABA levels. For the initial foliage ABA levels, three other random leaves were sampled to determine variation in the ABA levels in the plant (i.e. *n* = 6 leaves). The foliage ABA levels were assessed as described above. Reference lines regarding the minimum Ψ _*l*_ to trigger foliage ABA production consistent with the level measured under high VPD were determined using the relationship between ABA level and Ψ _*l*_, obtained from the ‘Foliage ABA accumulation’ data.

### VPD transitions of a wilty sunflower mutant

Individuals of wild-type sunflower cv. Argentario and the ABA-deficient mutant (*w-1*) of unknown genetic cause (Fambrini *et al.* 1995) were grown under similar conditions to well-watered cv. Yellow Empress plants. The VPD transitions were conducted by exposing similar aged (four-leaf stage) whole plants of both the wild type and *w-1* mutant to an increase in VPD in greenhouse conditions using a commercial dehumidifier. Plants were fully watered and bagged overnight to avoid ABA production before the beginning of the experiments. During the next day, VPD in the greenhouse was controlled using a dehumidifier at 1.0 kPa (± 0.2), one fully expanded leaf for each individual was enclosed in the 6-cm^2^ cuvette of a LI-6800 portable photosynthesis system (LICOR Inc., Lincoln, NE, USA). Initial conditions in the leaf cuvette were regulated at 25 °C, 390 μmol CO_2_ mol^−1^ air, PPFD of 1000 μmol m^−2^ s^−1^ and VPD of 1.0 ± 0.1 kPa. After measuring instantaneous leaf gas exchange (within 2 min of enclosure in the cuvette), leaf tissue was harvested for the quantification of ABA levels. The foliar ABA levels were quantified as described above. The VPD in the glasshouse was then increased to 2.0 kPa (± 0.25) and maintained for at least 60 min, the change in VPD took ~10 min. Instantaneous leaf gas exchange and foliar ABA levels were assessed on a neighbouring leaf after the step increase in high VPD.

### Statistical analysis

Differences in the turgor loss point and osmotic potential between sunflower plants grown under the two different conditions were tested by Student’s *t*-test (*n* = 3). Dynamic changes in the Ψ _*l*_ over the step increase in VPD for whole plants of sunflower from each growth condition were tested using one-way ANOVA (*n* = 3). Increases in foliage ABA levels of sunflower plants under low (*n* = 6) and high VPD (*n* = 3) were tested using paired Student’s *t*-test. Increases in foliage ABA levels in the wild type and *w-1* mutant from low to high VPD were tested using paired Student’s *t*-test (*n* = 4).

## Results

### Osmotic adjustment moves the threshold trigger for ABA biosynthesis

Growing sunflower plants under water-limited conditions outside, as opposed to well-watered conditions in a glasshouse, resulted in leaf osmotic adjustment, leading to both lower leaf osmotic potential and turgor loss point in these plants (Table 1). Leaf osmotic potential in water-limited plants was on average 0.45 MPa more than well-watered plants, while leaf turgor loss point was 0.33 MPa more negative than well-watered plants. This shift in the turgor loss point induced a consistent shift in the Ψ _*l*_ inducing major foliage ABA accumulation in bench-dried branches, which was always observed to occur close to the water potential at leaf turgor loss (Fig. 1).

**Table 1.** Mean (*n* = 3, ± SD) leaf osmotic potential at full turgor (Ψ _*s*_; MPa) and leaf water potential at turgor loss point (Ψ _tlp_; MPa) of sunflower cv. Yellow Empress plants grown under either well-watered or water-limited conditions. Asterisks denote significant changes (Student’s *t*-test; **P < 0.01, *P < 0.05) between growth conditions within species.

	Sunflower cv. Yellow Empress	
	Well-watered	Water-limited
Ψ _*s*_	−0.50 ± 0.02	−0.95 ± 0.09*
Ψ _tlp_	−0.71 ± 0.05	−1.04 ± 0.03**

### ABA is synthesized at high VPD if water potential drops below the threshold trigger

**Figure 2.**
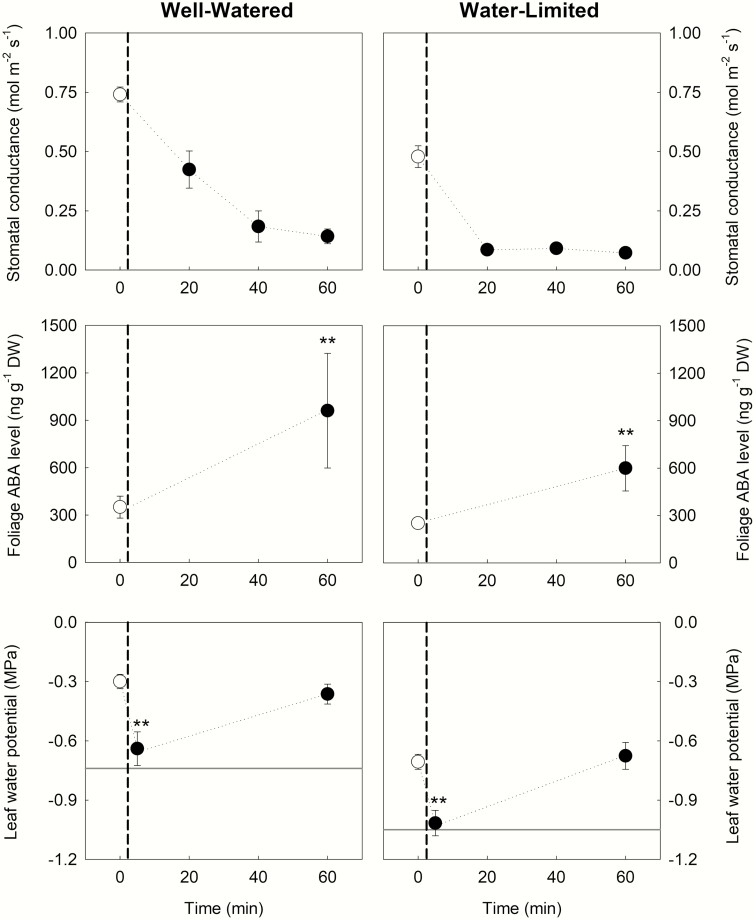
Mean response of instantaneous stomatal conductance, foliage ABA level and leaf water potential in sunflower cv. Yellow Empress after whole plants (*n* = 3, ± SD, and *n* = 6, ± SD for initial ABA level) were exposed to a step change in VPD from 0.75 kPa (white circles) to 3.25 kPa (black circles; change denoted by a vertical dashed line). The horizontal lines indicate the minimum leaf water potential necessary to trigger the accumulation of foliage ABA level in bench-dried branches (Fig. 1). Leaf water potential was measured 5 min after the VPD transition to capture the most negative leaf water potential values before stomatal began to close, no significant change in either stomatal conductance or ABA levels occurred within the first 5 min following the VPD transition (data not shown). Asterisks denote a significant change in leaf water potential among the three conditions or significant change in foliage ABA level compared with the initial one (Student’s *t*-test; **P < 0.01).

**Figure 3.**
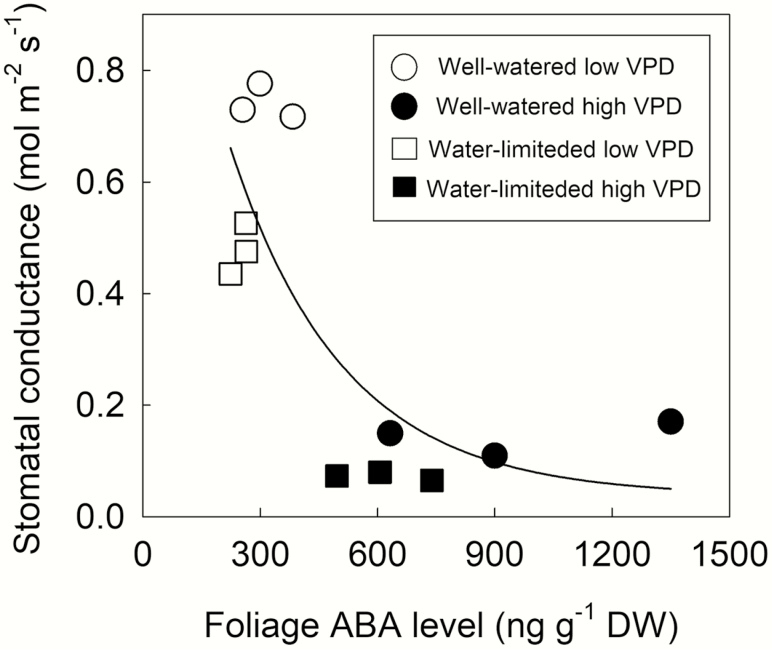
The relationship between instantaneous stomatal conductance and foliage ABA level in sunflower cv. Yellow Empress plants prior to (white symbols) and following (black symbols) a step change in VPD from 0.75 to 3.25 kPa, in plants grown under well-watered (circles) or water-limited (squares) conditions. *r*^2^ = 0.62, *P* < 0.05.

Similar physiological responses of sunflower plants grown under both well-watered and water-limited conditions were observed in response to a step increase in VPD imposed on the whole plant (Fig. 2). Stomatal closure was observed within the first 20 min of exposure to high VPD, stabilizing after 60 min. Foliage ABA levels increased after whole plants were exposed to the higher VPD. Ψ _*l*_ following the VPD transition rapidly declined to the threshold Ψ _*l*_ found to trigger the accumulation of foliage ABA levels in bench-dried branches (Fig. 1). At high VPD, Ψ _*l*_ was observed to relax back to the initial value measured at low VPD, presumably because of ABA-induced stomatal closure at high VPD after 60 min. During the VPD transitions for both well-watered and water-limited plants stomatal conductance strongly correlated with changes in foliage ABA level (*r*^2^ = 0.62, *P* < 0.05) (Fig. 3).

### ABA biosynthetic mutant plants do not have a stomatal response to increased VPD

**Figure 4.**
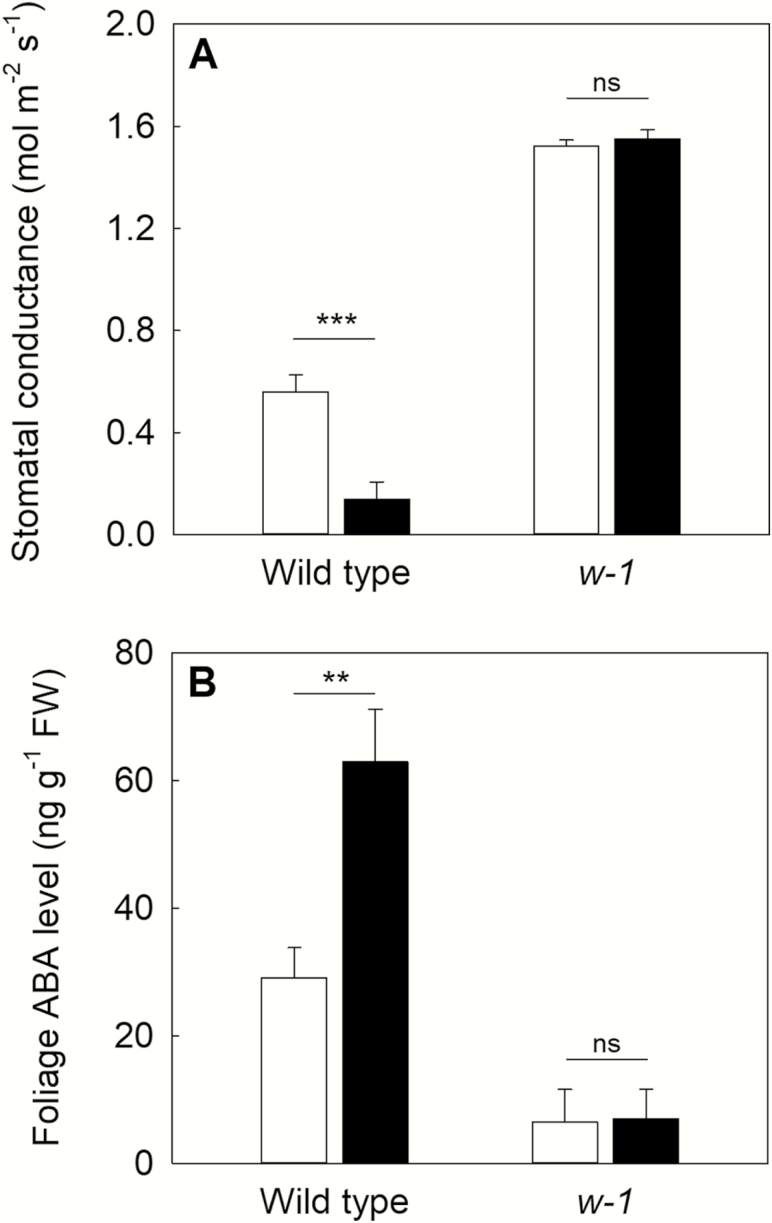
Mean steady-state stomatal conductance (A) and foliage ABA level (B) in plants (*n* = 4, ± SD) of sunflower cv. Argentario (wild type and *w-1* mutant) at a VPD of 1.0 kPa (white) and 2.0 kPa (black). Asterisks denote significant changes in foliage ABA levels under low and high VPD (Student’s *t*-test; ***P < 0.001, **P < 0.01, ns: not significant).

When wild-type plants of sunflower cv. Argentario were exposed to a moderate, step increase in VPD (from 1.0 to 2.0 kPa), stomata rapidly closed by 40 % (Fig. 4A). Consistent with this stomatal closure, an increase in the foliage ABA level was observed under high VPD in wild-type plants of sunflower cv. Argentario (Fig. 4B). In contrast, *w-1* mutant plants, which had more than double the initial stomatal conductance of wild-type cv. Argentario plants at low VPD (1.52 mol m^−2^ s^−1^ compared to 0.56 mol m^−2^ s^−1^), did not exhibit stomatal closure when exposed to the same increase in VPD (Fig. 4A). At the same time, the low foliage ABA level exhibits by the *w-1* mutant plants remained unchanged upon the step increase in VPD (Fig. 4B).

## Discussion

In this study, we present data that add to a growing body of evidence indicating that foliage ABA levels are major determinants of stomatal responses to VPD in angiosperms (Bauerle *et al.* 2004; Xie *et al.* 2006; Bauer *et al.* 2013; McAdam and Brodribb 2015, 2016; McAdam *et al.* 2016). First, we demonstrate that stomatal closure at high VPD occurs as Ψ _*l*_ transiently reaches values sufficiently low to result in the synthesis of foliage ABA levels in both control and osmotically adjusted plants of sunflower. Therefore, a strong association between foliage ABA level and stomatal conductance is found during VPD transitions in these plants. Second, we demonstrate contrasting stomatal responses to an increase in VPD between the wild-type Argentario and *w-1* ABA-deficient mutant (Fambrini *et al.* 1995), further indicating that ABA is critical for stomatal closure in sunflower plants exposed to high VPD. The lack of stomatal closure at high VPD in plants of *w-1* ABA-deficient mutant explains why these plants have a characteristically wilted appearance, with turgor pressure declining to zero on a daily basis despite growing in well-watered soil (Fambrini *et al.* 1994).

### Turgor loss point and foliage ABA accumulation

By bench-drying branches and using a high-precision method for ABA quantification, we indicate that Ψ _*l*_ at or near turgor loss point induces foliage ABA accumulation in sunflower in agreement with prevailing literature (Zabadal 1974; Beardsell and Cohen 1975; Pierce and Raschke 1980; Davies *et al.* 1981; Creelman and Zeevaart 1985). In addition to biosynthesis, the enhanced accumulation of ABA in these bench-dried leaves could be augmented by the inhibition of phloem export of ABA (Zeevaart and Boyer 1984). The consequence of adjustment in the threshold Ψ _*l*_ for ABA accumulation is that stomatal sensitivity to Ψ _*l*_ is shifted in osmotically adjusted plants. Here, we show that inducing changes in turgor loss point by osmotic adjustment results in consistent shifts in the Ψ _*l*_ trigger for the accumulation of ABA and consequently the Ψ _*l*_ at which stomata begin to close at high VPD. To our knowledge, no passive-hydraulic model for predicting stomatal responses to changes in VPD can account for this shift in sensitivity of stomata to Ψ _*l*_ driven by osmotic adjustment.

Whether changes in cell turgor (McAdam and Brodribb 2016), or changes in cell volume resulted from lowering Ψ _*l*_ (Sack *et al.* 2017) are the main signal responsible for upregulating the enzymes of the ABA biosynthetic pathway is yet to be resolved. There are a number of reasons to suggest that cell wall–cell membrane interactions, particularly at turgor loss point, are indeed the primary signal for ABA biosynthesis in leaves. Mutants defective in cuticle formation or biosynthesis appear similarly defective in ABA biosynthesis (Wang *et al.* 2011). Given that the cuticle provides structural rigidity for the leaf, constraining cell walls (Onoda *et al.* 2012), it is likely that alterations in turgor relations and not cell relative water content explain these observations. Furthermore, there are reports of unusual or deficient ABA biosynthesis in protoplasts exposed to osmotic solutions (Lahr and Raschke 1988; Bianco-Trinchant and Le Page-Degivry 1998), which cannot be explained if relative water content is the primary signal for ABA biosynthesis. Additional studies in this area, particularly the investigation of the genetic regulators of ABA biosynthesis, will hopefully resolve this critical unknown in plant biology. Our findings that ABA biosynthesis was only triggered in bench-dried shoots once dehydrated to turgor loss point suggests that a loss in cell turgor is the main trigger for ABA biosynthesis, as opposed to a decline in cell relative water content.

In sunflower, we observed a brief decrease in Ψ _*l*_ below the threshold trigger for ABA biosynthesis 5 min after the transition in VPD; however, by the time stomata had closed (20 min later) Ψ _*l*_ had relaxed to a value above the threshold trigger for ABA biosynthesis. In *Arabidopsis* a 5-min exposure of a leaf to positive pressure can trigger ABA biosynthesis (Sussmilch *et al.* 2017), suggesting that a similar period of time at which mesophyll cells are exposed to reduced cell volume or turgor can trigger ABA biosynthesis. Furthermore, the action of ABA on guard cells in leaves of the same species exposed to high VPD is known to occur within a similar time frame (Waadt *et al.* 2014), suggesting that ABA levels sufficient to cause stomatal closure can be produced rapidly on exposure to high VPD.

### ABA regulation of stomatal closure to high VPD

Our observations of stomatal closure at high VPD correlating with foliar ABA levels (Fig. 3) and the impaired stomatal response of *w-1*, a classical ABA-deficient mutant in sunflower (Fig. 4) are difficult to explain by the recent Merilo *et al.* (2018) model for stomatal regulation in angiosperms. According to this model an ABA-mediated, yet purely hydraulic, regulation of stomatal response to changes in VPD regulates stomatal responses to VPD in angiosperms (Merilo *et al.* 2018). This recent hypothesis suggests that ABA level sets steady-state stomatal conductance, and the ABA signalling pathway (not ABA levels *per se*) is responsible for dynamic stomatal closure in response to a step change in high VPD. The precise mechanism(s) behind the Merilo model remains obscure (Pantin and Blatt 2018). We found in sunflower, steady-state stomatal conductance in the ABA-deficient *w-1* mutant was higher than in the wild-type cv. Argentario, yet the stomata of this mutant did not close in response to high VPD. This is in agreement with previous studies in the mutant (Fambrini *et al.* 1995).

In *Populus* mutant plants in the ABA signalling pathway apparent stomatal conductance declines at high VPD, yet when corrected for intercellular vapour pressure stomatal conductance was found to remain unchanged in response to increasing VPD (Cernusak *et al.* 2019). It has been suggested that measurements of stomatal conductance, even in wild-type plants, are an underestimate (Vesala *et al.* 2017). The cause of reduced intercellular vapour pressure at high VPD in the ABA insensitive *Populus* mutants is likely due to evaporation through open stomata exceeding leaf hydraulic conductance. The lack of apparent stomatal closure as measured by gas exchange at high VPD in the *w-1* mutants of sunflower in this study suggests that leaves of this species have a high intrinsic leaf hydraulic conductance that is sufficient to supply the evaporative demand driven by open stomata at high VPD, unlike *Populus*. Further work in *Arabidopsis* is required to address the apparent reduction in stomatal conductance measured by leaf gas exchange in ABA biosynthetic mutants (Merilo *et al.* 2018). An alternative hypothesis, given recent controversy surrounding the validity of the conclusion that intercellular vapour pressure drops below saturation (Buckley and Sack 2019), is that stomatal closure in ABA-deficient mutants in *Arabidopsis* may be the result of an increased sensitivity to ABA, leading to stomatal closure in response to minor changes in the ABA levels in the single-gene ABA-deficient mutants (Szostkiewicz *et al.* 2010), and in ABA signalling mutants in *Populus*, genetic redundancy in the ABA signalling pathway (Leung *et al.* 1997).

We would conclude that the most parsimonious explanation for the stomatal responses to high VPD in sunflower is via the synthesis of ABA. Indeed, observations of wrong-way stomatal responses, which arise because of mechanical interactions between the guard cells and epidermal cells, add further complexity to explaining stomatal responses to VPD in angiosperms by purely passive-hydraulic mechanisms (Buckley 2019). A metabolic feedback signal is required to accurately predict right-way stomatal responses after wrong-way transients in angiosperms (Buckley 2015), and during VPD transitions (Schulze *et al.* 1974). We would argue that foliage ABA levels provide the best metabolic signal to explain this stomatal response in angiosperms. Although other metabolic signals, such as photosynthetic feedbacks, or combinations of other metabolic and passive signals (Granot *et al.* 2013; Salmon *et al.* 2020), or even unknown systemic signals (Ehonen *et al.* 2020), cannot be ruled out here without definitive molecular characterization of the *w-1* ABA-deficient mutant in sunflower.

## Supporting Information

The following additional information is available in the online version of this article—

**Figure S1.** Mean predawn (squares) and midday (circles) leaf water potentials over the course of a week observed in *Helianthus annuus* plants (*n* = 3, ± SD) grown under water-limited conditions.

**Table S1.** Summary of the main studies assessing stomatal closure of angiosperms under high leaf–air vapour pressure difference.

plaa025_suppl_Supplementary_Material
