# Supplementary material for: Osmotic adjustment and hormonal regulation of stomatal responses to vapour pressure deficit in sunflower
Source: AoB Plants. 2020 Jun 19;12(4):plaa025. doi: 10.1093/aobpla/plaa025 (PMC7346309; doi:10.1093/aobpla/plaa025)
Supplement: plaa025_suppl_Supplementary_Material [file plaa025_suppl_supplementary_material.docx]

**Supplementary Material**

**
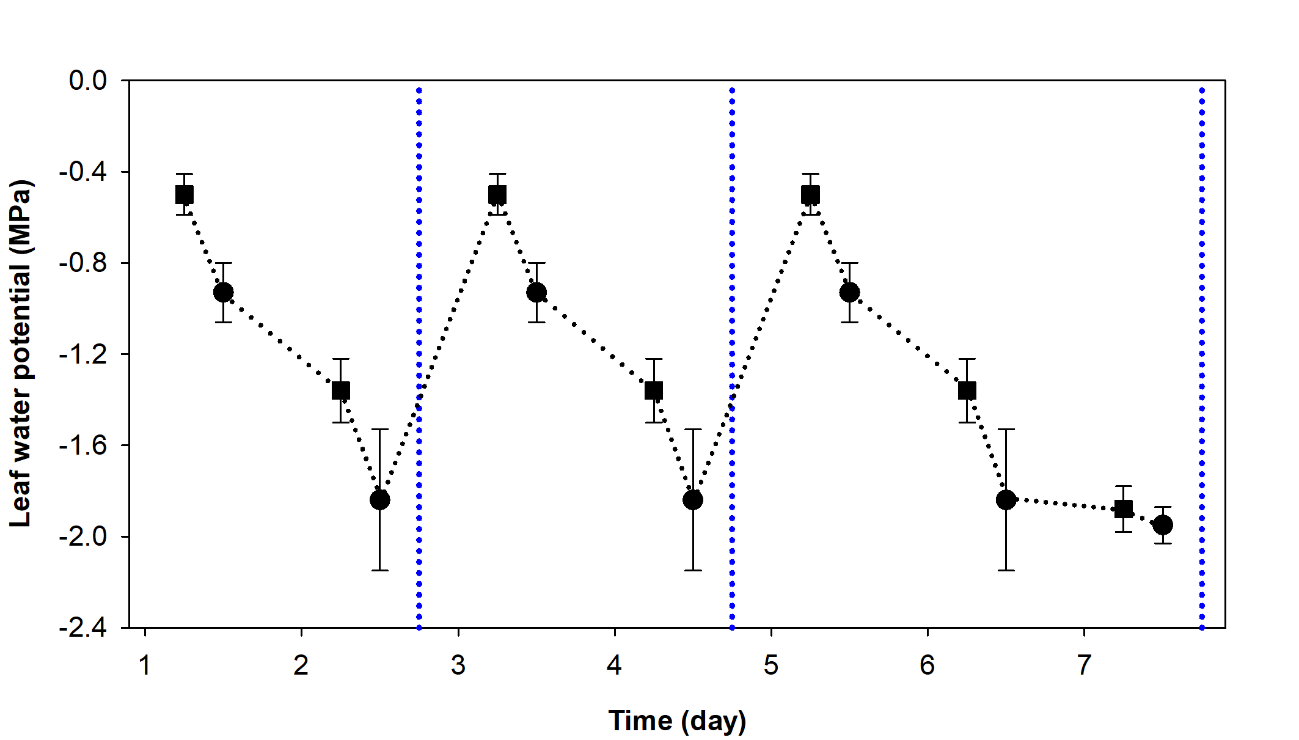
**

**Figure S1.** Mean predawn (squares) and midday (circles) leaf water potentials over the course of a week observed in *Helianthus annuus* plants (*n* = 3, ± SD) grown under water-limited conditions. Blue vertical lines indicate when plants were watered.

**Table S1**. Summary of the main studies assessing stomatal closure of angiosperms under high leaf-air vapor pressure difference (VPD). For each study, we provide the species used, whether stomatal closure in response to VPD was considered to be active or passive, the involvement of abscisic acid (ABA) and the proximal signal for stomatal closure.

| **Study** | **Species** | **Active or Passive** | **ABA involvement** | **Proximal signal** |
| --- | --- | --- | --- | --- |
| Schulze et al. (1974) | *Prunus armenica* | Active | Unknown | Leaf transpiration rate |
| Grantz (1990) | *Commelina communis* | Active | Potentially, in transpiration stream | Leaf transpiration rate |
|  | *Glycine max* |  |  |  |
|  | *Saccharum officinarum* |  |  |  |
| Mott and Parkhurst (1991) | *Glycine max*  *Nerium oleander*  *Phaseolus vulgaris*  *Vicia faba* | Passive | No | Leaf transpiration rate |
| Bunce (1996) | *Abutilon theophrasti* | Active | Yes, levels | Leaf transpiration rate and unknown signal |
|  | *Chenopodium album* |  |  |  |
|  | *Glycine max* |  |  |  |
| Assmann et al. (2000) | *Arabidopsis thaliana* | Passive | No | VPD |
| Xie et al. (2006) | *Arabidopsis thaliana* | Active | Yes, levels | Humidity |
| Bauerle et al. (2004) | *Acer rubrum* | Active | Yes, levels | VPD |
| Peak and Mott (2011) | *Tradescantia pallida* | Passive | No | Water vapor in substomatal cavity |
| Bauer et al. (2013) | *Arabidopsis thaliana* | Active | Yes, biosynthesis (guard cells) | Atmospheric humidity |
| Mott and Peak (2013) | *Nerium oleander*  *Pastinaca sativum*  *Xanthium strumarium* | Passive | No | Water vapor in substomatal cavity |
| McAdam and Brodribb (2015) | *Amborella trichopoda* | Active | Yes, levels | VPD |
|  | *Dahlia hybrida* |  |  |  |
|  | *Pisum sativum* |  |  |  |
|  | *Quercus robur* |  |  |  |
| McAdam et al. (2016) | *Arabidopsis thaliana* | Active | Yes, biosynthesis (leaves) | VPD |
|  | *Solanum lycopersicum* |  |  |  |
|  | *Pisum sativum* |  |  |  |
| McAdam and Brodribb (2016) | *Olea europaea* | Active | Yes, biosynthesis (leaves) | Mesophyll turgor |
|  | *Nothofagus cunninghamii* |  |  |  |
| Merilo et al. (2018) | *Arabidopsis thaliana* | Active and Passive | Yes, signaling pathway | VPD |
|  | *Solanum lycopersicum* |  |  |  |
|  | *Pisum sativum* |  |  |  |

**References**

Assmann SM, Snyder JA, Lee Y-RJ. 2000. ABA-deficient (aba1) and ABA-insensitive (abi1-1, abi2-1) mutants of Arabidopsis have a wild-type stomatal response to humidity. Plant, Cell and Environment 23:387–395

Bauer H, Ache P, Lautner S, Fromm J, Hartung W, Al-Rasheid Khaled AS, Sonnewald S, Sonnewald U, Kneitz S, Lachmann N, Mendel Ralf R, Bittner F, Hetherington Alistair M, Hedrich R. 2013. The stomatal response to reduced relative humidity requires guard cell-autonomous ABA synthesis. Current Biology 23: 53–57

Bauerle WL, Whitlow TH, Setter TL, Vermeylen FM. 2004. Abscisic acid synthesis in *Acer rubrum* L. leaves: a vapor-pressure-deficit-mediated response. Journal of the American Society for Horticultural Science 129, 182–187

Bunce JA. 1996. Does transpiration control stomatal responses to water vapour pressure deficit? Plant, Cell and Environment 20, 131–135

Grantz DA. 1990. Plant response to atmospheric humidity. Plant, Cell and Environment 13: 667–679

McAdam SAM, Brodribb TJ. 2015. The evolution of mechanisms driving the stomatal response to vapour pressure deficit. Plant Physiology 167: 833–843

McAdam SAM, Brodribb TJ. 2016. Linking turgor with ABA biosynthesis: implications for stomatal responses to vapor pressure deficit across land plants. Plant Physiology 171: 2008–2016

McAdam SAM, Sussmilch FC, Brodribb TJ. 2016. Stomatal responses to vapour pressure deficit are regulated by high speed gene expression in angiosperms. Plant, Cell and environment 39: 485–491

Merilo E, Yarmolinsky D, Jalakas P, Parik H, Tulva I, Rasulov B, Kilk K, Kollist H. 2018. Stomatal VPD response: there is more to the story than ABA. Plant Physiology 176, 851–864

Mott KA, Parkhurst DF. 1991. Stomatal responses to humidity in air and helox. Plant, Cell and Environment 14, 509–515

Mott KA, Peak D. 2013. Testing a vapour‐phase model of stomatal responses to humidity. Plant, Cell and Environment 36: 936–944

Peak D, Mott KA. 2011. A new, vapour‐phase mechanism for stomatal responses to humidity and temperature. Plant, Cell and Environment 34: 162–178

Schulze E-D, Lange OL, Evenari M, Kappen L, Buschbom U. 1974. The role of air humidity and leaf temperature in controlling stomatal resistance of *Prunus armeniaca* L. under desert conditions. Oecologia 17: 159–170

Xie X, Wang Y, Williamson L, Holroyd GH, Tagliavia C, Murchie E, Theobald J, Knight MR, Davies WJ, Leyser HMO, Hetherington AM. 2006. The identification of genes involved in the stomatal response to reduced atmospheric relative humidity. Current Biology 16: 882–887
